# Experiments of Image Classification Using Dissimilarity Spaces Built with Siamese Networks

**DOI:** 10.3390/s21051573

**Published:** 2021-02-24

**Authors:** Loris Nanni, Giovanni Minchio, Sheryl Brahnam, Gianluca Maguolo, Alessandra Lumini

**Affiliations:** 1 Department of Information Engineering (DEI), Via Gradenigo 6, 35131 Padova, Italy; giovanni.minchio@studenti.unipd.it (G.M.); gianluca.maguolo@phd.unipd.it (G.M.); 2 Department of Information Technology and Cybersecurity, Missouri State University, 901 S, National Street, Springfield, MO 65804, USA; sbrahnam@missouristate.edu; 3 Department of Computer Science and Engineering (DISI), University of Bologna, Via dell’Università 50, 47521 Cesena, Italy; alessandra.lumini@unibo.it

**Keywords:** audio sound classification, image classification, clustering, prototype selection, siamese network, dissimilarity space

## Abstract

Traditionally, classifiers are trained to predict patterns within a feature space. The image classification system presented here trains classifiers to predict patterns within a vector space by combining the dissimilarity spaces generated by a large set of Siamese Neural Networks (SNNs). A set of centroids from the patterns in the training data sets is calculated with supervised k-means clustering. The centroids are used to generate the dissimilarity space via the Siamese networks. The vector space descriptors are extracted by projecting patterns onto the similarity spaces, and SVMs classify an image by its dissimilarity vector. The versatility of the proposed approach in image classification is demonstrated by evaluating the system on different types of images across two domains: two medical data sets and two animal audio data sets with vocalizations represented as images (spectrograms). Results show that the proposed system’s performance competes competitively against the best-performing methods in the literature, obtaining state-of-the-art performance on one of the medical data sets, and does so without ad-hoc optimization of the clustering methods on the tested data sets.

## 1. Introduction

The most common image classification strategy involves extracting features from samples and then training classifiers to discriminate them within the selected feature space. Another less common method involves training patterns within one or more (dis)similarity spaces. The idea of (dis)similarity, or semblance, is grounded in human learning and plays a fundamental role in theories of knowledge and behavior [1]. For this reason, (dis)similarity provides a sound theoretical basis for building learning algorithms. Training in (dis)similarity spaces is considered particularly relevant when addressing large multiclass problems [2] and when samples have discernible patterns, as is often the case when dealing with shapes, spectra, images, and texts [3]. The basic idea of (dis)similarity classification is to estimate an unknown sample’s class label based on the similarities/dissimilarities between the sample and a set of labeled training samples and pairwise (dis)similarities between the training samples. Some simple (dis)similarity measures popular in computer vision include the tangent distance [4], earth mover’s distance (EMD) [5], shape matching distance [6], and the pyramid match kernel [7]. Because classification within (dis)similarity spaces does not require access to a sample’s features, the sample space can be any set and not limited to the Euclidean space as long as the (dis)similarity function is well defined for any pair of samples [8]. 

Dissimilarity spaces can be defined by pairwise dissimilarities computed between complex objects like images, audio, time signals, spectra, graphs [9], 3D data, and in all problems where a distance measure between target objects can be specified more naturally than can a feature representation [3]. The feature space is substituted by a proximity-based representation space (RS) in which general-purpose classifiers are trained on all training objects that demand comparisons to a small set of prototypes. The RS space can be generated according to any meaningful dissimilarity measures, including non-Euclidean and nonmetric ones [10].

One line of research in dissimilarity spaces focuses on developing different approaches for defining an RS space. The two most common are direct learning with similarity functions [11] and kernel methods [12]. It is also worth noting that some researchers have conducted extensive experimentation on dissimilarity-based classifiers, comparing them with traditional feature-based classifiers and concluding that this classification scheme outperforms traditional classifiers in a large set of applications, thereby indicating that these classifiers have a separate domain of competence [13]. 

Rather than selecting a predefined distance measure beforehand, a distance metric can be learned during training. This is a process known as Metric Learning (MeL). A general framework for MeL is proposed in [14], which the authors call Adaptive Nearest Neighbor and which is experimentally demonstrated to produce a broader search space within which better solutions can be found. Of recent note is a hybrid meta-learning model called Meta-Metric-Learner [15] that can handle flexible numbers of classes and generate generalized metrics for classification across domains. Other recent developments involve the application of deep learning for MeL [16,17,18,19]. In [19], for example, the authors developed a General Pair Weighting (GPW) framework that transforms the sampling problem of deep metric learning into a unified view of pair weighting through gradient analysis. In [20], a metric learning approach makes use of a Siamese Neural Network (SNN) [21] to minimize and maximize the distance between pairs of images. For a survey of deep MeL, see [22]. 

Before moving on, it is important to clarify terms. As pointed out in [23], the terms *distance* and *(dis)similarity* are often used interchangeably in the literature, but *(dis)similarity* is the broader term in that it can be produced by a range of functions that are not distance measures. In other words, (dis)similarity can be viewed not only as a distance within a space but also as a means for building other spaces. Moreover, though at first, it might appear that the choice to distinguish two objects based on either their similarities or dissimilarities is arbitrary (the terms *similarity* and *dissimilarity* are often used interchangeably in the literature), the type of data and the problem itself have a bearing on the selection of one perspective over the other [23].

The focus in this paper, as indicated by the title, is on image classification based on dissimilarities, an idea introduced in [3], where differences are considered between samples of different classes. Dissimilarity approaches can be divided into two types, those based on dissimilarity vectors [24] and those on dissimilarity spaces [25], a nomenclature that was introduced in [23]. Dissimilarity vectors transform a multiclass problem into a two-class problem by computing the difference between feature vectors extracted from two samples. If the two samples belong to the same class, they are considered positive; else they are deemed negative. The basic idea is for the classifier to distinguish whether a dissimilarity vector was generated from samples that either belong or do not belong to the same class. This method was introduced in [24]. Some work based on [24] includes [26] and [27], where both papers propose the idea of combining classifiers using receiver operating characteristic (ROC). In [28], handcrafted texture features, such as scale-invariant feature transform (SIFT), speeded up robust features (SURF), and local binary patterns (LBP) and its variants, were used to generate a set of classifiers on the dissimilarity space. Explored as well was the impact of dynamic classifier selection strategies. In [29], the authors reduced sensitivity to a large number of classes in auditory bird species identification by combining the extraction of features from audio spectrograms with the dissimilarity vector approach. Finally, in [30], features extracted from convolutional neural networks (CNNs) were combined via the dissimilarity vector approach. 

Dissimilarity methods based on dissimilarity spaces derive classifiers from feature vector spaces where a vector represents the distance between pairs of samples compared to the classical feature space where a feature vector represents a sample as measured over all features. For instance, in [31], the authors used prototype selection to develop classifiers based on dissimilarity spaces, and the dissimilarity representations were treated as a vector space. In [32], a strategy for learning dissimilarity for interactive image retrieval was proposed. Following the method described in [25], dissimilarity was adjusted via a prototype-based dissimilarity space. In [33], descriptors were combined to capture the gradient and textural characteristics of patterns using sparse representation in the dissimilarity space. 

More recently, researchers have begun to define dissimilarity spaces generated by deep learners. For example, in [34], a dissimilarity space was built on top of deep convolutional features, which produced a compact representation based on prototype selection methods. In addition, MeL methods were used in the dissimilarity space rather than the Euclidean distance. In [35], the authors proposed a variant that works well for the dissimilarity representation space of the common maximum mean discrepancy (MMD) loss. The MMD variant aligns the source and target data in the dissimilarity space by exploiting the structure of intra-class and inter-class distributions, in this way producing a domain-invariant pairwise matcher. In [36], the authors modified the traditional contrastive loss function of the Siamese network to create a distance model learned by training SNN on dissimilarity values for brain image classification; the system works by predicting the correlation distance between the output features of image pairs. Finally, in [37] and [38], systems for audio classification were developed by expanding the dissimilarity methods proposed in [36]. Dissimilarity spaces were generated by a set of clustering techniques and a small set of SNNs with different backbones. The clustering methods transformed the audio images (spectrograms) in a bird [39] and a cat [40,41] vocalization data set into a set of centroids that generated the dissimilarity space through the twin networks. Each audio pattern was then projected into these spaces to obtain a vector space representation that was fed into an SVM. The system was shown to produce superior results compared to the standalone CNNs.

The system proposed in this work extends and generalizes the audio classification systems developed in [37] and [38] with the goal of producing not only a more powerful system but also one that can handle different types of images, not just audio spectrograms. To accomplish this goal, the new system is built with a large set of eight different CNN architectures selected for the twin classifiers, with four new CNN architectures presented here. Heterogeneous auto-similarities of characteristics (HASC) [42] features are extracted from the aforementioned bird [39] and cat [40,41] data sets as well as on a medical data set for classifying narrow-band imaging (NBI) endoscopic videos [43] and a data set of images for the classification of the maturation of human stem cell-derived retinal pigmented epithelium [44]. In the training phase, a clustering algorithm is employed to select a set of *relevant* samples to be used as the prototypes of the training samples. Moreover, a distance measure is inferred by training a set of SNNs for comparing pairs of samples. In the testing phase, an unknown pattern is compared to the centroids (prototypes) of the dissimilarity spaces generated by the set of SNNs in order to measure the dissimilarity of two patterns. In this fashion, the dissimilarity spaces represent each input pattern (consisting of both the original images and the images processed by HASC) by a feature vector obtained by calculating its distances from each of the centroids. Decisions are based on a fusion by sum rule of the SVMs trained on the vectors generated by the different dissimilarity spaces (produced by changing the value of *k* in the clustering methods) and by the different network topologies. The proposed image classification system (produced without ad-hoc optimization of the clustering methods on the tested data sets) is compared to the state-of-the-art as well as with fusions with the state-of-the-art. Results demonstrate the generalizability and power and of this approach, which achieved similar results on the audio and the medical data to the best performing methods reported in the literature and state-of-the-art performance on one of the medical data sets.

The remainder of this paper is organized as follows. In Section 2, an outline of the proposed system is provided that, for clarity, considers only one SNN. In Section 3, all eight SNN backbones used to generate the dissimilarity spaces are described in detail with a focus on the four new backbones used in this work. In Section 4, the clustering methods are presented. In Section 5, experimental results are provided and discussed, along with some comparisons on the same data sets with other classifier systems. The paper concludes in Section 6 with some suggestions for future work.

## 2. Proposed System

**Figure 1:**
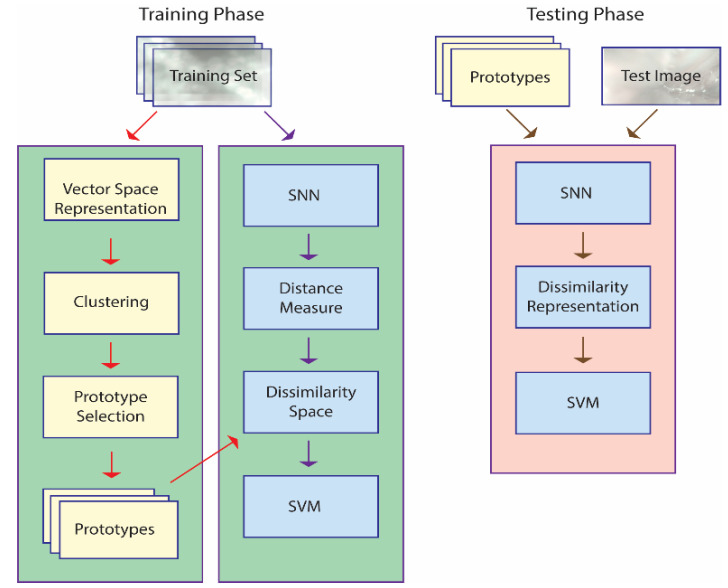
A basic outline of the proposed approach. Note: in the training phase, prototype selection is performed, and an SNN is trained to define a dissimilarity measure; in the testing phase, each unknown pattern is represented by its distances to the prototypes and classified accordingly.

An illustration of the approach taken in this work is provided in Figure 1, which outlines the basic steps taken using only one SNN, though a set of eight is combined in the whole system. The main steps outlined in Figure 1 are explained in more detail in the subsections that follow. Algorithms in pseudocode are available for each step in [37] and [38], and the MATLAB source code used in this work is available at https://github.com/LorisNanni accessed on 20 January 2021.

The training phase is geared towards generating a dissimilarity space via an SNN that learns a distance measure d(x,y) from a set of prototypes $P\;=\;p_{1},\;\ldots p_{k}$. The SNN is trained to maximize the dissimilarity between pairs of images belonging to one class while at the same time minimizing the dissimilarity for pairs of images belonging to all the other classes. The set of prototypes are the k centroids of the clusters produced by k-means applied to a vector space representation of the images in the training set. The end result is a feature vector $f\;\in \;R^{k}$ that represents image x in the dissimilarity space, where for a given $f_{i}$ the distance between x and the prototype is $p_{i}:\;f_{i}\;=\;d\left(x,\;p_{i}\right)$. This feature vector is used to train an SVM. 

The testing phase represents an unknown pattern by projecting it onto a dissimilarity space. The feature vector is obtained by calculating the pattern’s distance to the set of prototypes, P. This feature vector is fed into the SVM to determine its class. Both the original images in the data sets and the HASC [42] descriptors (outlined in Section 2.5) serve as the input to the classification process. 

### 2.1. SNN Training

To generate the dissimilarity space, the SNN is trained to compare two images and return a dissimilarity value where larger values indicate that the images belong to the same class and smaller values mean that both images belong to different classes. Details regarding the eight SNN architectures are provided in Section 3.

### 2.2. Prototype Selection

To reduce the dimensionality of the dissimilarity space, prototype selection is accomplished by extracting from the training set only *k* prototypes using the supervised k-means clustering technique outlined in Section 4. Without dimensionality reduction, it would be too difficult to maintain each training sample as a prototype. 

### 2.3. Projection in the Dissimilarity Space

To predict patterns by projecting them into a dissimilarity space, as proposed here, each pattern x is characterized by its dissimilarity to a set of prototypes $P\;=\;p_{1},\;\ldots p_{k}$ and by the dissimilarity feature vector F defined as the dissimilarity of pattern d(x, y) as given by a trained SNN:

(1) $$F\left(x\right)=\;\left[d\left(x,\;p_{1}\right),\ldots ,d\left(x,\;p_{i}\right),,\ldots ,\;d\left(x,p_{k}\right)\right].\;$$

Input patterns are compared with the *k* prototypes (stored in *P*) via the distance measure learned by the SNN. The number of centroids is a parameter that is determined by testing a set of values for k that are dependent on the number of classes c: k=kc× c, kc={15, 30 45, 60}. The feature space *F* is the output that includes the projections of all the input images.

### 2.4. SVM Classification

SVM [45] is a classic learner that searches for a hyperplane that separates data belonging to two classes. Prediction is a matter of mapping an unseen pattern to the side of the hyperplane that represents its class. If the data are not linearly separable, kernel functions can be employed to map the data into higher-dimensional spaces where the data can be separated. SVM can handle multilabel problems by training an ensemble of SVMs and then by combining their decisions using a one-against-all method that classifies a pattern as belonging to the class with the highest confidence score. Such is the approach taken here.

### 2.5. HASC

HASC [42] is a local descriptor designed to capture the linear covariances (COV) and nonlinear entropy combined with mutual information (EMI) relational characteristics of an object. Some of the advantages of covariance matrices as descriptors include their low dimension, robustness to noise, and their ability to capture the features of the joint PDF. Covariance matrices suffer from two main disadvantages, however. First, outlier pixels can make these descriptors more sensitive to noise; and, second, these descriptors can only encapsulate the features of the joint PDF when the features are linked by a linear relation. HASC overcomes these limitations by combining COV with EMI. The entropy (E) of EMI is a measurement of a random variable’s uncertainty, while the mutual information (MI) of two random variables captures generic dependencies: both linear and nonlinear. The modeling of both linear and nonlinear dependencies is what makes HASC a robust descriptor.

HASC descriptors are extracted by dividing an image into patches and generating the EMI matrix (d×d). The main diagonal of EMI encapsulates the unpredictability (E) of the d features. The off-diagonal (element i,j)  captures the mutual dependency (MI) between the i-th and j-th feature. HASC is computed by concatenating the vectorized form of EMI and COV.

The MI of a pair of random variables A,B is calculated as:

(2) $$MI\left(A,B\right)={\int^{\text{​}}}_{A}{\int^{\text{​}}}_{B}\;p\left(a,b\right)\log\left(\frac{p\left(a,b\right)}{p\left(a\right)p\left(b\right)}\right)dbda,\;$$

where p(a), p(b), and p(a,b) are the PDF of A, the PDF of B, and their joint PDF, respectively.

In the case where A=B, then MI is the entropy of A:

(3) $$E\left(A\right)=\;MI\left(A,A\right)=-{\int^{\text{​}}}_{A}p\left(a\right)\log\left(p\left(a\right)\right)da.$$

If there exists a finite set M  of realization pairs, then MI can be estimated as a sample mean inside the logarithm:

(4) $$MI\left(A,B\right)\approx \frac{1}{M}\sum_{m=1}^{M}\log\left(\frac{p\left(a_{k},b_{k}\right)}{p\left(a_{k}\right)p\left(b_{k}\right)}\right).$$

A fast way to calculate the probabilities from the M realizations inside the logarithm is to estimate them by building a joint 2D normalized histogram of values A and B, such that $p\left(a_{k},b_{k}\right)$ is estimated by taking the value of the 2D histogram bin containing the pair $a_{k},b_{k}$. In this fashion, $p\left(a_{k}\right)$ and $p\left(b_{k}\right)$ can be estimated by summing all the bins corresponding to $a_{k}$ and $b_{k}$, respectively, and the i,j-th components of EMI related to the patch P. Thus, EMI can be calculated as:

(5) $$EMI_{p\left\{ij\right\}}=\frac{1}{M}\sum_{m=1}^{N}\log\left(\frac{\tilde{p}\left(z_{mi},z_{mj}\right)}{\tilde{p}\left(z_{mi}\right)\tilde{p}\left(z_{mj}\right)}\right),$$

where $\tilde{p}$(…) and $\tilde{p}$(.) are the probabilities estimated with the histogram, and $z_{mi}$ is the i-th feature at pixel M.

For this study, HASC is extracted from the whole image. The output FEAT of the function HASC is a three-dimensional matrix (w × h × d) that contains all the features extracted from the whole image. The dimension d is the number of low-level features. The number of bins in the histograms in Equation (5) is 28, and the number of low-level features is 6 (these are the default parameters). FEAT is reshaped to construct the vector img= [FEAT (:,:,1) FEAT (:,:,2); FEAT (:,:,3) FEAT (:,:,4); FEAT (:,:,5) FEAT (:,:,6)], and this vector is resized to serve as input to a CNN.

## 3. Siamese Neural Network (SNN)

**Figure 2:**
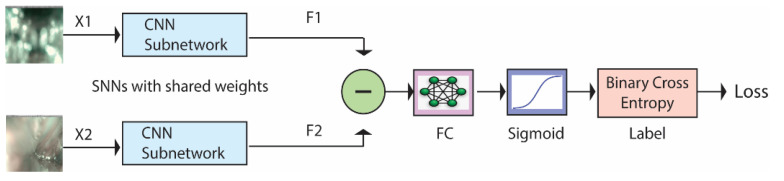
Siamese Neural Network architecture.

SNNs are a class of deep architectures that take two images as input and compute the dissimilarity between them [21]. SNNs are called *Siamese networks* because they are made by combining two identical subnetworks whose outputs are subtracted and fed into a fully connected layer. Figure 2 illustrates how these networks work. They are trained to recognize whether the two input images (X1 and X2) belong to the same class or not. The CNN subnetworks produce feature vectors (F1 and F2) of size 2048 or 4096. The subtract block, FC Layer, and sigmoid function are described in Section 3.2. The binary cross-entropy gives the loss function between the predicted score and the true label value. A more detailed description of SNNs can be found in [46].

### 3.1. The Two Identical Twin Subnetworks

In this study, eight backbone networks are used in the Siamese architectures. In Table 1, the sequence of the CNN layers is reported. 

The subnetworks use two different activation functions. The first one is ReLU [47], and the other is leaky ReLU [48], which is a modification of ReLU, defined as:

(6) $$y_{i}=\;f\left(x_{i}\right)=\{\begin{array}{c} 0,\;\;x_{i}<0\\ x_{i},\;\;x_{i}\geq 0 \end{array}$$

Leaky ReLU is an activation function like ReLU that is equivalent to the identity function for positive values but has a hyperparameter α>0  for negative inputs, guarantying that the gradient of the activating function is never zero so that the optimization process is less likely to become stuck in local minima. Leaky ReLU, however, alleviates problems caused by the hard zero activations of ReLU. 

Leaky ReLU is defined as:

(7) $$y_{i}=\;f\left(x_{i}\right)=\{\begin{array}{c} ax_{i},\;\;x_{i}<0\\ x_{i},\;\;x_{i}\geq 0, \end{array}$$

where a  is a real number (a=1  here).

Table 1 describes the SNN backbones. The strategy in designing the topologies was to start from a well-established and simple architecture and gradually vary the internal layers (variations were informed by bibliographical suggestions and practitioner experience), with the main aim of obtaining diversity in the final classification results. The backbones listed in Table 1 are the result of a preliminary trial-and-error phase, where nine topologies were tested and trained on the first fold on the Bird data set. Only those networks that converged on the training data are reported below. Network 1 is the simple baseline convolutional topology suggested by MATLAB for a Siamese network. The other topologies are designed by adding variations to this baseline. Network 2 is an architecture that uses leaky ReLU. In Network 3, the nonlinearities are alternated using either ReLU or max pooling after every convolutional layer. Network 4 is similar to Network 1 but has different hyperparameters. In Network 5, the sequence of layers reduces the size of the hidden layers to be very low before the last FC layers. Hence, it has few parameters since the FC layer is small. Network 6 is the deepest network, with the size of the hidden layers decreasing very smoothly. Network 7 has a dropout layer immediately after the input layer. In addition, it has no ReLU layers, and all the nonlinearities are pooling layers. Network 8 is the shallowest network. However, it is the one with the largest number of parameters since the last FC layer is the largest.

### 3.2. Subtract Block, FC Layer, and Sigmoid Function

As illustrated in Figure 2, the subtract block operation subtracts the output of the two networks and computes the absolute value, returning the feature vector:

(8) Y = |F1 − F2|.

Notice that, thanks to the absolute value, this quantity is unchanged by switching the inputs X1 and X2, which is consistent with the fact that the similarity of two samples should be a symmetric function. The FC layer and the sigmoid function learn to predict the dissimilarity of the inputs. The dissimilarity measure is not a metric since it does not satisfy the triangular inequality and the identity property. However, it is continuous with respect to the Euclidean metric, which means that arbitrarily small changes in the input size lead to arbitrarily small changes in the output.

## 4. Clustering

Clustering algorithms segregate unlabeled samples into groups that maximize the similarity and differences between members. Most of these algorithms calculate a centroid (the mean) during the clustering process. Because centroids capture the salient characteristics of patterns within a cluster, they can help reduce the dimensionality of the dissimilarity space without losing too much critical information. Increasing the number of centroids within each class retains even more significant information.

K-means clustering is one of the most popular and simplest clustering algorithms and is the method used here. It partitions samples into *k* clusters defined apriori by placing each observation into a cluster based on the nearest centroid as measured by the Euclidean Distance. The standard k-means algorithm is a four-step process:

Step 1. Randomly select a set of centroids from the training data points;
Step 2. For each remaining data point in the training set, find the distance between it and the nearest centroid;
Step 3. Calculate new centroids via a weighted probability distribution;
Step 4. Repeat Steps 2 and 3 until convergence.

## 5. Results

The generic image classification system proposed here is tested and compared with the standalone classifiers and the state-of-the-art using four data sets representing two classification tasks: audio classification (bird and cat vocalizations), with audio represented by spectrograms, and two medical data sets (endoscopic videos and image-based classification of maturation of human stem cell-derived retinal pigmented epithelium). The testing protocol used for each data set is that which was initially proposed in the original papers. The performance indicator is classification accuracy. The three data sets are described and labeled in the experiments as follows:

- BIRDz [39]: This balanced data set is a real-world benchmark for bird species vocalizations. The testing protocol is ten-runs using the data split in [39]. The audio tracks were extracted from the Xeno-Canto Archive (http://www.xeno-canto.org/ accessed on 20 January 2021). BIRDz contains a total of 2762 acoustic samples from eleven North American bird species along with 339 unclassified audio samples (consisting of noise and unknown bird vocalizations). The bird classes vary in size from 246 to 259. Each observation is represented by five spectrograms: (1) constant frequency, (2) frequency modulated whistles, (3) broadband pulses, (4) broadband with varying frequency components, and (5) strong harmonics;
- CAT [40,41]: This data set has ten balanced classes of cat vocalizations, with each class containing ~300 samples for a total of 2962 samples taken from Kaggle, Youtube, and Flickr. The testing protocol is 10-fold cross-validation. The average duration of each sample is 4 s.
- InfLar [43]: This data set contains eighteen narrow-band imaging (NBI) endoscopic videos of eighteen different patients with laryngeal cancer. The videos were retrospectively analyzed and categorized into four classes based on quality of the images (informative, blurred, with saliva or specular reflections, and underexposed). The average video length is 39s. The videos were acquired with an NBI endoscopic system (Olympus Visera Elite S190 video processor and an ENF-VH rhino-laryngo videoscope) with a frame rate of 25 fps and an image size of 1920 × 1072 pixels. A total of 720 video frames, 180 for each of the four classes was extracted and labeled. The testing protocol is three-fold cross-validation with data separated at the patient level to ensure that the frames from the same class were classified based on the features characteristic of each class and not on features linked to the individual patient (e.g., vocal fold anatomy).
- RPE [44]: This is a data set that contains 195 images for the classification of maturation of human stem cell-derived retinal pigmented epithelium. The images were divided into sixteen subwindows, each of which was assigned to one of four classes: (1) Fusifors (216 images of nuclei and separated cells that are fuse shaped), (2) Epithelioid (547 images of relatively packed cells and nuclei that are globular in shape), (3) Cobblestone (949 images of well-defined cell contours and cell walls that are tightly packed, homogeneous cytoplasm, and hexagonal in shape), and (4) Mixed (150 images containing two or more instances of the other three classes). Removed were images that were out of focus or that contained only background information or other clutter. The resulting total number of labeled images is 1862.

The Siamese networks in our experiments were trained with the options suggested by the MATLAB framework for Siamese networks to make sure the values were not overfitted on the selected data set. The parameters for ADAM optimization are learning rate: 0.0001; gradient decay factor: 0.9; and squared gradient decay factor: 0.99. The number of iterations was set to 3000 with no stop criterion.

The performance measures selected for evaluating the proposed approach and for comparison with the literature are Area Under the ROC-curve (AUC) and accuracy. Both are commonly reported in image classification. Accuracy is the ratio of the number of true positives and the number of examples in the testing set. AUC is an indicator applied to two-class problems and expresses the probability a given learner will assign a higher score to a randomly picked positive sample versus a randomly picked negative one [49]. The “one vs. all” method for calculating a multiclass AUC is reported in the experiments presented here.

The ensembles listed in Table 2 and Table 3 were obtained by varying the network topology and the input data (Sp refers to the spectrograms in the audio data sets; Im to the original images in the InLar data set, and HASC to HASC features restructured as images). The clustering method is k-means for all methods, and the number of prototypes belongs to the set {15, 30, 45, 60}. The column **#classifiers** provides the number of classifiers in the ensemble, and the first column **Name** is the label assigned to the ensemble.

As shown in Table 2 and Table 3, the best average performance is obtained by the ensemble F_NN6/8 using HASC images as the inputs to the Siamese network. Combining by sum rule F_NN6-HASC and F_NN6-Spect/Im, the performance on CAT is 85.08, on BIRD 94.92, and on InfLar 87.64. Clearly, the ensembles strongly outperform the network topologies. The superiority of one method over another can be validated with the Wilcoxon signed-rank test [50]: F_NN6-Hasc outperforms each of the other methods (except F_NN8-Hasc) with a *p*-value of 0.05.

The performance of the methods in [37,38] on the InfLar/RPE data sets is calculated in this work using the original code, with no variation. 

It was shown in [38] that making ensembles of Siamese networks by varying clustering algorithms is not as advantageous as combining different topologies. For this reason, in this work, the focus is only on generating ensembles of Siamese networks trained with different topologies. Reported in Table 4 and Table 5 is a comparison between the Siamese networks and standard CNNs tested in previous papers. The CNN labeled eCNN is the sum rule among the different CNNs tested in a given data set. Accuracy is reported in Table 4 and AUC in Table 5. The following conclusions can be drawn examining Table 4 and Table 5:

- The proposed F_NN6-Hasc ensemble improves previous methods based on Siamese networks;
- F_NN6 obtains a performance that is similar to eCNN on BIRD but lower than eCNN on the other data sets;
- Results show that the gap in performance between an ensemble of Siamese networks and CNNs is closing.

The best performance across all four data sets is obtained by the weighted sum rule between eCNN and F_NN6/8-Hasc (i.e., the fusion of the CNNs and the Siamese networks). Before the fusion, the scores of eCNN and F_NN6/8-Hasc were normalized to mean 0 and standard deviation 1. In the weighted sum rule, the weight of eCNN is 4 (since we use 4 CNNs), while the weight of F_NN6/8-Hasc is 1.

The fine-tuning of CNN pre-trained on ImageNet on the data sets is reported in Table 4 and was performed with the following training options: batch size: 30; max epoch: 20; learning rate: 0.0001 (for all the networks with no freezing). Data augmentation was applied only for InfLar with image reflections on the two axes and random rescaling using a factor uniformly sampled in [1,2]. No data augmentation was used for CAT and BIRD, where the input is a spectrogram. Moreover, it should be stressed that no data augmentation to reduce computation time was used with the Siamese networks.

GoogleNet was also trained with the HASC images. In this case, performance dropped compared to training on the original images. Also tested was ResNet50 as a backbone for the Siamese networks, but it failed to converge in our tests.

In Table 6, the state-of-the-art is reported on the tested data sets using the same testing protocols that were used in all the other experiments. The performance of the ensembles presented in this paper approximate those reported in the literature and obtain the state-of-the-art performance on the InfLar data set. This shows the generalizability and power of the proposed system. In the RPE data set, the fusion of Siamese and CNNs does not improve eCNN, but Hasc clearly improves performance on that data set. 

Note that in Table 6 two results are reported from [40]; they are distinguished with the labels [40] and [40]*−CNN*.

For a fairer comparison among the different topologies, a fuller experimental evaluation across many more image/video data sets is required. Be that as it may, we believe that the experiments presented in this paper speak to the robustness and generalizability of the proposed system, which achieves competitive classification accuracy compared to the state-of-the-art in the literature across four different image data sets without any ad-hoc parameter tuning. Moreover, results were obtained following a clear and unambiguous testing protocol. The value of reporting the results of a system across different data sets is that the results can reasonably serve as a baseline for comparisons with new methods introduced in the future.

## 6. Conclusions

The image classification system proposed here experimentally derived an ensemble of Siamese networks that were utilized to generate dissimilarity spaces for the purpose of image classification. A compact descriptor was obtained by projecting each sample into the dissimilarity spaces generated by k-means using different sets of centroids combined with the outputs of a set of eight Siamese networks. The classification step was performed by SVMs trained on the resulting descriptors, with the SVMs combined by sum rule. Both the original images and HASC images served as the input. This approach resulted in a highly competitive ensemble, as tested on four very different data sets: two for animal vocalization classification, one for classifying narrow-band imaging (NBI) endoscopic videos, and another for classifying the maturation of human stem cell-derived retinal pigmented epithelium. Experimental results demonstrated the competitiveness and generalizability of the proposed approach compared to other methods, with the new system achieving the state-of-the-art on the InfLar NBI video data set. The fusions improved performance on all four data sets, outperforming the standalone CNNs.

Future work generating dissimilarity spaces with Siamese networks will focus on experimentally deriving more robust and generalizable ensembles. The goal will be to assess this approach across many more classification problems, such as those cited in [36,56]. 

## Figures and Tables

**Table 1:** CNN Siamese Networks (1–8) layers.

**Siamese Network 1**				
**Layers**	**Activations**	**Learnable**	**Filter Size**	**Num. of Filters**
Input Layer	224 × 224			
2D Convolution	215 × 215 × 64	6464	10 × 10	64
ReLU	215 × 215 × 64	0		
Max Pooling	107 × 107 × 64	0	2 × 2	
2D Convolution	101 × 101 × 128	401,536	7 × 7	128
ReLU	101 × 101 × 128	0		
Max Pooling	50 × 50 × 128	0	2 × 2	
2D Convolution	47 × 47 × 128	262,272	4 × 4	128
ReLU	47 × 47 × 128	0		
Max Pooling	23 × 23 × 128	0	2 *×* 2	
2D Convolution	19 × 19 × 64	204,864	5 *×* 5	64
ReLU	19 × 19 × 64	0		
Fully Connected	4096	94,638,080		
**Siamese Network 2**				
**Layers**	**Activations**	**Learnable**	**Filter Size**	**Num. of Filters**
Input Layer	224 × 224	0		
2D Convolution	220 × 220 × 64	1664	5 *×* 5	64
LeakyReLU	220 × 220 × 64	0		
2D Convolution	216 × 216 × 64	102,464	5 *×* 5	64
LeakyReLU	216 × 216 × 64	0		
Max Pooling	108 × 108 × 64	0	2 *×* 2	
2D Convolution	106 × 106 × 128	73,856	3 *×* 3	128
LeakyReLU	106 × 106 × 128	0		
2D Convolution	104 × 104 × 128	147,584	3 *×* 3	128
LeakyReLU	104 × 104 × 128	0		
Max Pooling	52 × 52 × 128	0	2 *×* 2	
2D Convolution	49 × 49 × 128	262,272	4 *×* 4	128
LeakyReLU	49 × 49 × 128	0		
Max Pooling	24 × 24 × 128	0	2 *×* 2	
2D Convolution	20 × 20 × 64	204,864	5 *×* 5	64
LeakyReLU	20 × 20 × 64	0	5 *×* 5	
Fully Connected	2048	52,430,848		
**Siamese Network 3**				
**Layers**	**Activations**	**Learnable**	**Filter Size**	**Num**. **Filters**
Input Layer	224 × 224			
2D Convolution	55 × 55 × 128	6400	7 *×* 7	128
Max Pooling	27 × 27 × 128	0	2 *×* 2	
2D Convolution	23 × 23 × 256	819,456	5 *×* 5	256
ReLU	23 × 23 × 256	0		
2D Convolution	19 × 19 × 128	819,328	5 *×* 5	128
Max Pooling	9 × 9 × 128	0	2 *×* 2	
2D Convolution	7 × 7 × 64	73,792	3 *×* 3	64
ReLU	7 × 7 × 64	0		
Max Pooling	3 × 3 × 64	0	2 *×* 2	
Fully Connected	4096	2,363,392		
**Siamese Network 4**				
**Layers**	**Activations**	**Learnable**	**Filter Size**	**Num. of Filters**
Input Layer	224 × 224			
2D Convolution	218 × 218 × 128	6400	7 *×* 7	128
Max Pooling	54 × 54 × 128	0	4 *×* 4	
ReLU	54 × 54 × 128	0		
2D Convolution	50 × 50 × 256	819,456	5 *×* 5	256
ReLU	50 × 50 × 256	0		
2D Convolution	48 × 48 × 64	147,520	3 *×* 3	64
Max Pooling	24 × 24 × 64	0	2 *×* 2	
2D Convolution	22 × 22 × 128	73,856	3 *×* 3	128
ReLU	22 × 22 × 128	0		
2D Convolution	18 × 18 × 64	204,864	5 *×* 5	64
Fully Connected	4096	84,938,752		
**Siamese Network 5**				
**Layers**	**Activations**	**Learnable**	**Filter Size**	**Num**. **of Filters**
Input Layer	224 × 224			
2D Convolution	215 × 215 × 64	6464	10 *×* 10	64
Max Pooling	107 × 107 × 64	0	2 *×* 2	
ReLU	107 × 107 × 64	0		
2D Convolution	26 × 26 × 128	401,536	7 *×* 7	128
ReLU	26 × 26 × 128	0		
2D Convolution	9 × 9 × 128	409,728	5 *×* 5	128
ReLU	9 × 9 × 128	0		
2D Convolution	6 × 6 × 64	131,136	4 *×* 4	64
ReLU	6 × 6 × 64	0		
Fully Connected	4096	9,441,280		
**Siamese Network 6**				
**Layers**	**Activations**	**Learnable**	**Filter Size**	**Num**. **of Filters**
Input Layer	224 × 224			
2D Convolution	218 × 218 × 64	3200	7 *×* 7	64
Max Pooling	109 × 109 × 64	0	2 *×* 2	
ReLU	109 × 109 × 64	0		
2D Convolution	107 × 107 × 128	73,856	3 *×* 3	128
Max Pooling	53 × 53 × 128	0	2 *×* 2	
ReLU	53 × 53 × 128	0		
2D Convolution	53 × 53 × 64	8256	1 *×* 1	64
ReLU	53 × 53 × 64	0		
2D Convolution	51 × 51 × 128	73,856	3 *×* 3	128
ReLU	51 × 51 × 128	0		
Max Pooling	25 × 25 × 128	0	2 *×* 2	
2D Convolution	25 × 25 × 128	16,512	1 *×* 1	128
ReLU	25 × 25 × 128	0		
2D Convolution	22 × 22 × 64	131,136	4 *×* 4	64
Max Pooling	11 × 11 × 64	0	2 *×* 2	
ReLU	11 × 11 × 64	0		
Fully Connected	4096	31,723,520		
**Siamese Network** 7				
**Layers**	**Activations**	**Learnable**	**Filter Size**	**Num. of Filters**
Input Layer	224 × 224			
Dropout Layer	224 × 224	0		
2D Convolution	218 × 218 × 64	3200	7 *×* 7	64
Max Pooling	109 × 109 × 64	0	2 *×* 2	
2D Convolution	105 × 105 × 128	204,928	5 *×* 5	128
Max Pooling	52 × 52 × 128	0	2 *×* 2	
2D Convolution	48 × 48 × 64	204,864	5 *×* 5	64
Max Pooling	24 × 24 × 64	0	2 *×* 2	
2D Convolution	22 × 22 × 256	147,712	3 *×* 3	256
Max Pooling	11 × 11 × 256	0	2 *×* 2	
2D Convolution	9 × 9 × 256	590,080	3 *×* 3	256
Fully Connected	4096	16,781,312		
**Siamese Network 8**				
**Layers**	**Activations**	**Learnable**	**Filter Size**	**Num. of Filters**
Input Layer	224 × 224			
2D Convolution	215 × 215 × 32	3232	10 *×* 10	32
Max Pooling	107 × 107 × 32	0	2 *×* 2	
ReLU	107 × 107 × 32	0		
2D Grouped Convolution	101 × 101 × 64	50,240	7 *×* 7	64
2D Convolution	97 × 97 × 128	204,928	5 *×* 5	128
Max Pooling	48 × 48 × 128	0	2 *×* 2	
ReLU	48 × 48 × 128	0		
2D Grouped Convolution	46 × 46 × 256	147,712	3 *×* 3	256
Fully Connected	4096	2.218,790,912		

**Table 2:** Performance accuracy obtained considering different network topologies with HASC input images. Best values are in bold-faced.

Name	Input Image	Network Topology	#Classifiers	CAT	InfLar	BIRD	RPE
	HASC	NN1	4	78.64	90.56	94.52	84.46
	HASC	NN2	4	81.69	88.33	93.22	84.75
	HASC	NN3	4	78.64	79.44	94.91	82.59
	HASC	NN4	4	82.37	88.33	93.33	84.58
	HASC	NN5	4	78.98	87.64	94.04	80.09
	HASC	NN6	4	80.68	89.72	93.09	85.22
	HASC	NN7	4	76.61	80.97	91.97	82.18
	HASC	NN8	4	78.64	85.69	91.37	80.84
F_NN4	HASC	NN1 … NN4	16	84.07	89.86	94.99	84.80
F_NN6	HASC	NN1 … NN6	24	84.41	**91.11**	**95.10**	**85.24**
F_NN8	HASC	NN1 … NN8	32	**84.75**	90.56	**95.10**	84.80
[37]				82.41	74.86	92.97	66.19
[38]				84.07	89.86	94.99	84.80

**Table 3:** Performance accuracy obtained by different network topologies with input image = Spect/Im (note: to reduce computation time and considering the low performance of RPE on Spect/IM (the SNNs do not always converge during training), we only report the performance of NN1 and NN2). Best values are in bold-faced.

Name	Input Image	Network Topology	#Classifiers	CAT	InfLar	BIRD	RPE
	Spect/Im	NN1	4	78.64	74.72	92.46	63.60
	Spect/Im	NN2	4	76.95	71.39	92.74	37.81
	Spect/Im	NN3	4	75.25	83.47	93.02	---
	Spect/Im	NN4	4	81.36	74.17	91.86	---
	Spect/Im	NN5	4	76.95	81.25	94.03	---
	Spect/Im	NN6	4	78.31	75.46	91.96	---
	Spect/Im	NN7	4	72.54	66.81	88.43	---
	Spect/Im	NN8	4	79.32	77.92	94.14	---
F_NN4	Spect/Im	NN1 … NN4	16	79.32	79.17	93.44	---
F_NN6	Spect/Im	NN1 … NN6	24	81.69	80.69	93.76	---
F_NN8	Spect/Im	NN1 … NN8	32	83.39	79.58	94.24	---

**Table 4:** Performance accuracy obtained considering different standard CNN. Best values are in bold-faced.

Method	CAT	BIRD	InfLar	RPE
F_NN4-Hasc	84.07	94.99	89.86	84.80
F_NN6-Hasc	84.41	95.10	91.10	85.24
F_NN8-Hasc	84.75	95.10	90.56	84.00
GoogleNet	82.98	92.41	90.42	87.70
VGG16	84.07	95.30	91.53	89.27
VGG19	83.05	95.19	92.22	89.30
GoogleNetP365	85.15	92.94	93.61	88.51
eCNN	87.36	95.81	94.03	89.82
F_NN6-Hasc + eCNN	**88.14**	**96.04**	**95.56**	89.75
F_NN8-Hasc + eCNN	**88.14**	**96.04**	94.86	**89.86**

**Table 5:** Performance (AUC) obtained considering different standard CNNs. Best values are in bold-faced.

Method	CAT	BIRD	InfLar	RPE
[37]	0.967	0.983	0.906	
F_NN4-Hasc	0.973	0.993	0.982	0.938
F_NN6-Hasc	0.973	0.993	0.985	0.937
F_NN8-Hasc	0.975	0.995	0.985	0.933
GoogleNet	0.979	0.994	0.992	0.966
VGG16	0.984	**0.997**	0.994	0.966
VGG19	0.981	**0.997**	0.995	**0.972**
GoogleNetP365	0.986	0.995	0.993	0.969
eCNN	**0.987**	**0.997**	0.996	**0.972**
F_NN6-Hasc + eCNN	0.986	0.996	**0.997**	0.968
F_NN8-Hasc + eCNN	**0.987**	**0.997**	**0.997**	0.969

**Table 6:** Literature results (accuracy).

Authors	Reference	CAT	BIRD	InfLar	RPE
Nanni et al.	[51]	—	96.3	—	—
Nanni et al.	[52]	—	95.1	—	—
Zhao et al.	[53]	—	93.6	—	—
Pandeya & Lee.	[41]	87.7	—	—	—
Pandeya et al.	[40]	91.1	—	—	—
Pandeya et al.	[40]*−CNN*	90.8	—	—	—
Zhang et al.	[39]	—	96.7	—	—
Patrini et al.	[54]	—	—	93.25	—
Moccia et al.	[43]	—	—	80.25	—
Nanni et al.	[55]	—	—	—	97.1
